# Janus kinase inhibitors ameliorate clinical symptoms in patients with STAT3 gain-of-function

**DOI:** 10.1093/immadv/ltad027

**Published:** 2023-11-24

**Authors:** Shuya Kaneko, Fumiaki Sakura, Kay Tanita, Asami Shimbo, Ryusuke Nambu, Masashi Yoshida, Shuichiro Umetsu, Ayano Inui, Chizuru Okada, Miyuki Tsumura, Mamiko Yamada, Hisato Suzuki, Kenjiro Kosaki, Osamu Ohara, Masaki Shimizu, Tomohiro Morio, Satoshi Okada, Hirokazu Kanegane

**Affiliations:** Department of Pediatrics and Developmental Biology, Graduate School of Medical and Dental Sciences, Tokyo Medical and Dental University (TMDU), Tokyo, Japan; Department of Pediatrics, Hiroshima University Graduate School of Biomedical and Health Sciences, Hiroshima, Japan; Department of Pediatrics and Developmental Biology, Graduate School of Medical and Dental Sciences, Tokyo Medical and Dental University (TMDU), Tokyo, Japan; Department of Pediatrics and Developmental Biology, Graduate School of Medical and Dental Sciences, Tokyo Medical and Dental University (TMDU), Tokyo, Japan; Division of Gastroenterology and Hepatology, Saitama Children’s Medical Center, Saitama, Japan; Division of Gastroenterology and Hepatology, Saitama Children’s Medical Center, Saitama, Japan; Department of Pediatric Hepatology and Gastroenterology, Saiseikai Yokohama-shi Tobu Hospital, Kanagawa, Japan; Department of Pediatric Hepatology and Gastroenterology, Saiseikai Yokohama-shi Tobu Hospital, Kanagawa, Japan; Hiroshima Chuodori Children Clinic, Hiroshima, Japan; Department of Pediatrics, Hiroshima University Graduate School of Biomedical and Health Sciences, Hiroshima, Japan; Center for Medical Genetics, Keio University School of Medicine, Tokyo, Japan; Center for Medical Genetics, Keio University School of Medicine, Tokyo, Japan; Center for Medical Genetics, Keio University School of Medicine, Tokyo, Japan; Department of Technology Development, Kazusa DNA Research Institute, Chiba, Japan; Department of Pediatrics and Developmental Biology, Graduate School of Medical and Dental Sciences, Tokyo Medical and Dental University (TMDU), Tokyo, Japan; Department of Pediatrics and Developmental Biology, Graduate School of Medical and Dental Sciences, Tokyo Medical and Dental University (TMDU), Tokyo, Japan; Department of Pediatrics, Hiroshima University Graduate School of Biomedical and Health Sciences, Hiroshima, Japan; Department of Child Health and Development, Graduate School of Medical and Dental Sciences, Tokyo Medical and Dental University, Tokyo, Japan

**Keywords:** signal transducer and activator of transcription 3, gain-of-function, Janus kinase inhibitor, tofacitinib, ruxolitinib, lymphoblastoid cell lines

## Abstract

Germline gain-of-function (GOF) variants in the *signal transducer and activator of transcription 3* (*STAT3*) gene is an inborn error of immunity presenting with autoimmunity and lymphoproliferation. Symptoms can vary widely, and no effective treatment has been established. This study investigated the efficacy of Janus kinase (JAK) inhibitors (JAKi) in patients with STAT3-GOF. Four patients were enrolled and their clinical symptoms before and after the initiation of treatment with JAKi were described. A cell stimulation assay was performed using Epstein-Barr virus transformed lymphoid cell lines (EBV-LCLs) that were derived from the patients with STAT3-GOF. The patients presented with various symptoms, and these symptoms mostly improved after the initiation of JAKi treatment. Upon interleukin-6 stimulation, the EBV-LCLs of patients showed enhanced STAT3 phosphorylation compared with those of the EBV-LCLs of healthy controls. In conclusion, four Japanese patients with STAT3-GOF were successfully treated with JAKi. JAKi ameliorated various symptoms and therefore, the use of JAKi could be an effective treatment option for patients with STAT3-GOF.

## Graphical Abstract

**Graphical Abstract:**
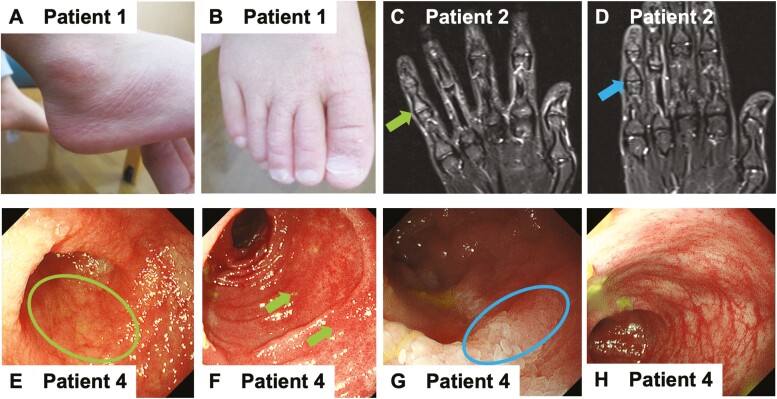

## Introduction

Signal transducer and activator of transcription 3 (STAT3) is a protein that regulates cell differentiation and proliferation through the activation of signal transduction and transcription [1]. In the cytokine signaling cascade, STAT3 is tyrosine-phosphorylated by Janus kinase (JAK) and translocated as a dimer into the nucleus, where it activates transcription [2].

Germline gain-of-function (GOF) variants in the *STAT3* (STAT3-GOF) gene cause early-onset immune dysregulation syndrome, which is characterized by lymphoproliferation and multi-organ autoimmunity, including type 1 diabetes mellitus, polyarthritis, inflammatory bowel disease (IBD), and interstitial pneumonia (IP) [3–7]. Interestingly, the pattern of abnormal immunity in patients with STAT3-GOF is inconsistent and the clinical features vary widely among patients.

Previously, we reported on seven Japanese patients from five families harboring germline heterozygous activating *STAT3* variants and their clinical and immunological features [8]. They presented with various symptoms; however, no effective treatment had been established at that time.

Recently, the incidence of patients with STAT3-GOF has increased worldwide, and the efficacy of immunosuppressive drugs and targeted treatments, such as monoclonal antibodies against the interleukin-6 (IL-6) receptor and JAK inhibitors (JAKi), has been reported [4, 9–13]. However, the sample size remains small with only a few studies confirming treatment efficacy *in vitro*, and most of these studies reported clinical efficacy.

In this study, we describe the clinical efficacy of JAKi targeted treatment in four Japanese patients with STAT3-GOF, including one newly diagnosed case. In addition, we confirmed that JAKi suppressed STAT3 phosphorylation *in vitro*, and these results support the clinical improvements observed in the various autoimmune symptoms of patients with STAT3-GOF.

## Patients and methods

### Study approval

This study was conducted in accordance with the recommendations of the Declaration of Helsinki and was approved by the Ethics Committee of Tokyo Medical and Dental University. The committee’s reference number is G2019-004. Interventions such as JAKi were given during the routine clinical care of patients, and therefore the study was not registered as a clinical trial.

### Western blot analysis

Epstein–Barr virus transformed lymphoid cell lines (EBV-LCLs) that were derived from the patients before JAKi initiation were stimulated with 40 ng/ml of fusion protein of IL-6R and IL-6 (FP6) (R&D systems, Minneapolis, MN, USA) with and without 1.0 μM tofacitinib citrate (TOF) (Adipogen life science, San Diego, CA, USA) or with and without 1.0 μM ruxolitinib (RUXO) (Cayman Chemical, Ann Arbor, MI, USA) for 15 min and subjected to western blot analysis. Equal amounts of proteins were separated by 10% sodium dodecyl sulfate-polyacrylamide gel electrophoresis (SDS–PAGE) and transferred to polyvinylidene fluoride membranes (PVDF; Merck KGaA, Darmstadt, Germany). The membranes were blocked with PVDF blocking agent (TOYOBO, Tokyo, Japan) or low-fat bovine milk and incubated with the primary antibodies rabbit anti-human pSTAT3 (pY705) antibody (Cell Signaling Technology, Danvers, MA, USA), mouse anti-human STAT3 antibody (Cell Signaling Technology), or rat anti-human GAPDH antibody (BioLegend, San Diego, CA, USA) diluted with Immunoreaction Enhancer Solution (TOYOBO), or low-fat bovine milk. The horseradish peroxidase (HRP)-conjugated goat anti-mouse, anti-rabbit (Thermo Fisher Scientific, MA, USA), and anti-rat antibodies (GE Healthcare, Buckinghamshire, UK) were used as secondary antibodies. Antibody binding was detected using an enhanced chemiluminescence reagent (Cytiva Amersham, Amersham, UK). Band intensity quantification was measured using ImageJ software 1.53a (National Institutes of Health, Bethesda, MD, USA), and the mean values were shown.

### Statistical analysis

Statistical analyses were performed using GraphPad Prism 8 (GraphPad, San Diego, CA, USA). The results are expressed as the means. The between-group differences were assessed using the two-tailed non-parametric Mann–Whitney *U*-test. *P*-values of <0.05 were considered statistically significant.

## Results

### Case presentation

**Figure 1.**
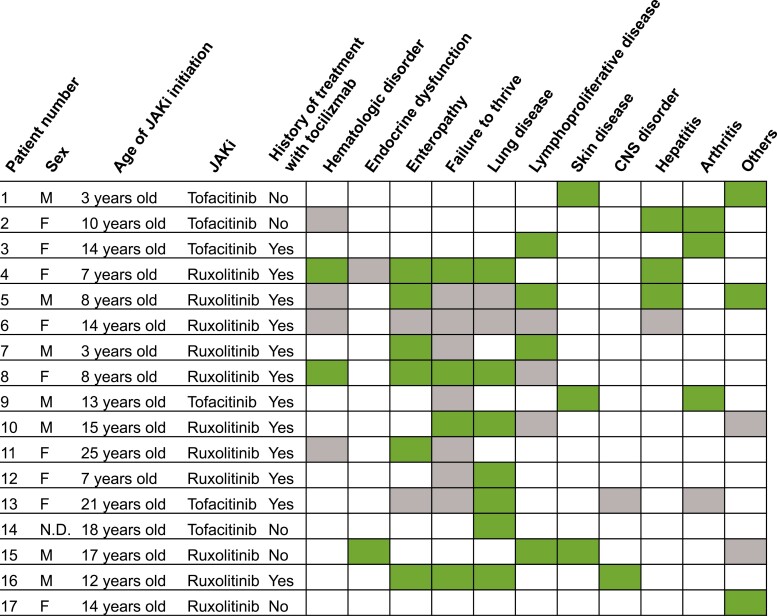
**Clinical features of our patients and a literature review of previously reported STAT3-GOF patients treated with JAK inhibitors.** Patients No. 1–4 are in the patient 1–4 of this study, Patients No. 5–10 are in the patient 12–17 of the study by Forbes et al. [11], No. 11 is in the report of Parlato et al. [10], Patient No. 12–14 are in the patient 1, 2, and 4 in the study by Silva-Carmona et al. [14], Patient No. 15 is in the report by Wegehaupt et al. [15], Patient No. 16 is the report by Sarfati et al. [16], and Patient No. 17 is in the report by Mulvihill et al. [13]. Symptoms highlighted in green cells are complete or partially responded symptoms. Symptoms highlighted in gray cells are not responded symptoms or not described for the responses in the reports. CNS, central nervous system; JAK, Janus kinase; JAKi, JAK inhibitors; N.D., No data.

The patient’s clinical information is shown in Figure 1 and Supplementary Table 1, and the patient’s symptoms when receiving the JAKi are underlined (Supplementary Table 1).

**Figure 2.**
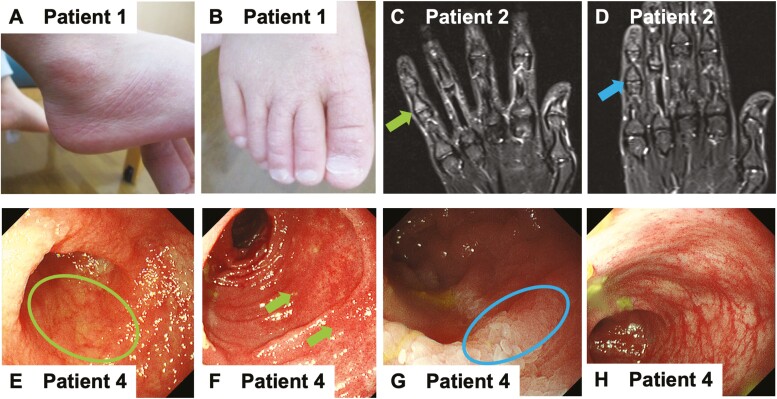
**Clinical images of the patients.** (A and B) Images of the right ankle and toes of patient 1 before the administration of tofacitinib. The patient had an atopic dermatitis-like skin rash. (C and D) Magnetic resonance imaging findings of the right hand of patient 2 before and after the initiation of tofacitinib treatment. (C) Findings before starting tofacitinib treatment with an enhanced T2-weighted short tau inversion recovery image around the proximal interpharangeal joint of the little finger which is suggestive of arthritis, including bone marrow edema and contrast effects of the tendon (green arrow). (D) Findings 6 months after initiation of tofacitinib treatment. Abnormal lesions showed improvement (blue arrow). (E–H) Endoscopic images of patient 4 before and after the administration of ruxolitinib. (E and G) Terminal ileum; (F and H) Descending colon; (E and F) Before administration of ruxolitinib treatment; (G and H) After 4 months of ruxolitinib treatment. Initially, the ileum showed total villous atrophy (green circle) and the colon showed a decreased vascular pattern with edematous mucosa (green allows); however, after the administration of ruxolitinib these abnormal findings showed improvement (blue circle).

**Figure 3.**
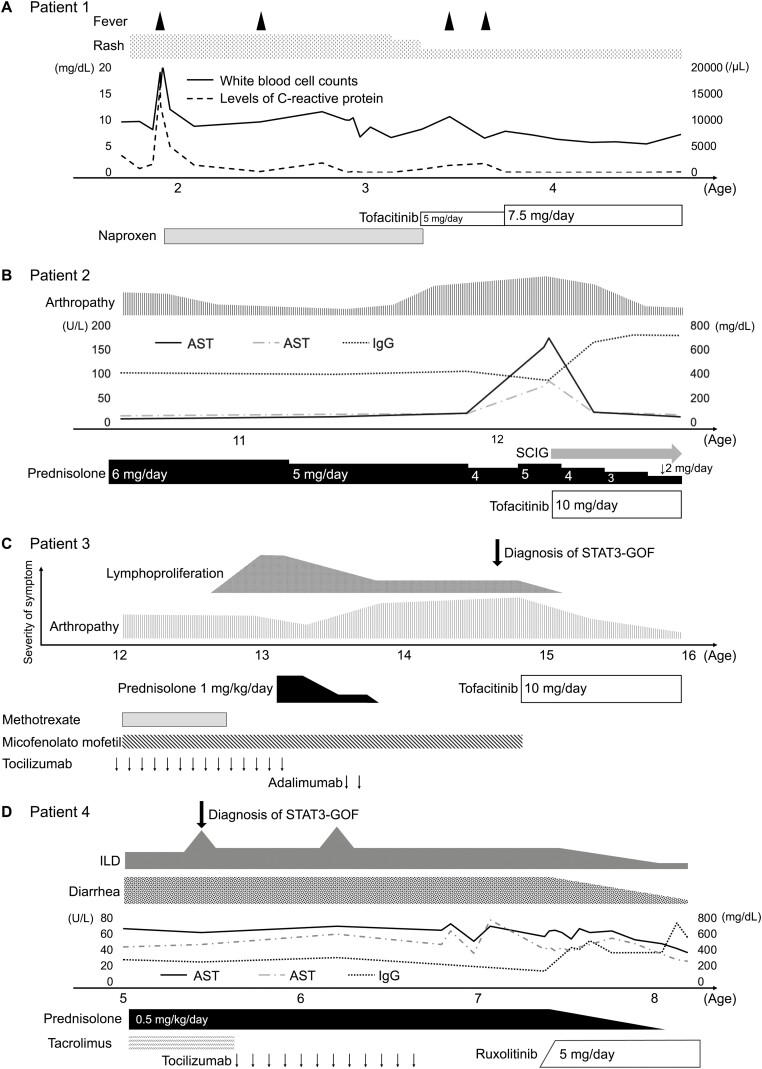
**Clinical course of patients before and after initiation of JAK inhibitor treatment.** (A) Patient 1 (p.K348E) was a 17-month-old boy that had a recurrent fever of unknown origin combined with an atopic dermatitis-like skin rash. (B) Patient 2 (p.G618A) was an 8-year-old girl that presented with arthropathy and autoimmune hepatitis. (C) Patient 3 (p.P715L) was a 14-year-old girl that presented with refractory polyarthritis and lymphoproliferation. (D) Patient 4 (p.E415G) was a 4-year-old girl that presented with refractory diarrhea, severe failure to thrive, and interstitial pneumonia. ALT, alanine aminotransferase; AST, aspartate aminotransferase; IgG, immunoglobulin G; SCIG, subcutaneous immunoglobulin.

Patient 1 (p.K348E) was a 17-month-old boy and was identical to P2 of our previous report [8]. He had a fever of unknown origin, signs of inflammation combined with an atopic dermatitis-like skin rash (Fig. 2A and B), and cervical lymphadenopathy. He received antibiotics and intravenous immunoglobulin for incomplete Kawasaki disease. However, his fever was recurrent. He was diagnosed with STAT3-GOF, and TOF 5 mg/day was initiated at the age of 3 years. After TOF initiation, although his skin rash improved instantaneously, his intermittent fevers were recurrent, and his C-reactive protein (CRP) levels remained mildly elevated. The TOF dose was increased to 7.5 mg/day, after which he had no further recurrent fevers and his CRP level normalized (Fig. 3A).

Patient 2 (p.G618A) was an 8-year-old girl and was identical to P4.1 of our previous report [8]. She presented with a stomachache and positive fecal occult blood at the age of 6 years. Further medical evaluation revealed autoimmune hepatitis, unclassified IBD, and multiple enthesitis of the wrist and ankle. She also experienced vitiligo and had mild hypogammaglobulinemia (IgG, 500–600 mg/dl). Because of the enthesitis of the wrist and liver dysfunction, it was challenging to reduce the prednisolone dose. She received 10 mg/day TOF at the age of 12. After TOF initiation, her arthralgia and liver dysfunction improved and the dose of prednisolone was reduced (Fig. 3B).

Patient 3 (p.P715L) was a 14-year-old girl and a new patient who was not included in our previous report. She presented with refractory polyarthritis since the age of 1 year. At the age of 4 years, she was diagnosed with polyarticular juvenile idiopathic arthritis, and immunosuppressive agents including prednisolone, methotrexate, and tocilizumab were administered. These drugs were effective; however, remission was only partially achieved for a short duration. At the age of 8 years, she experienced autoimmune neutropenia and thrombocytopenia. At the age of 14 years, she was referred to our department because of refractory polyarthritis and lymphadenopathy of unknown cause. She was diagnosed with STAT3-GOF and received 10 mg/day TOF. After TOF initiation, her arthralgia improved, and through magnetic resonance imaging, it could be seen that the bone marrow edema of the fingers disappeared (Figs. 2C, D, and 3C).

Patient 4 (p.E415G) was a 4-year-old girl and was identical to P3 of our previous report [8]. She had severe viral and bacterial infections since early infancy. She had refractory diarrhea and severe failure to thrive requiring total parenteral nutrition from birth. Since the age of 2 years, she had hypothyroidism, type 1 diabetes mellitus, IP, and chronic liver disease. Administration of immunosuppressive agents including steroids, azathioprine, tacrolimus, and tocilizumab resulted in short-term partial remission. She occasionally received extracorporeal membrane oxygenation due to IP exacerbations, and parenteral nutrition therapy had been introduced due to severe autoimmune enteropathy (anti-villin antibody-positive). Endoscopy revealed total villous atrophy in her ileum and duodenum, and a decreased vascular pattern and edematous mucosa in her colon (Fig. 2E and F). She received RUXO at the age of 8 years. RUXO contributed to the alleviation of enteropathy, IP, and chronic liver disease (Figs. 2G and H, and 3D). Unfortunately, she died at the age of 8 years, which was unrelated to the STAT3-GOF symptoms.

### Western blot analysis

**Figure 4.**
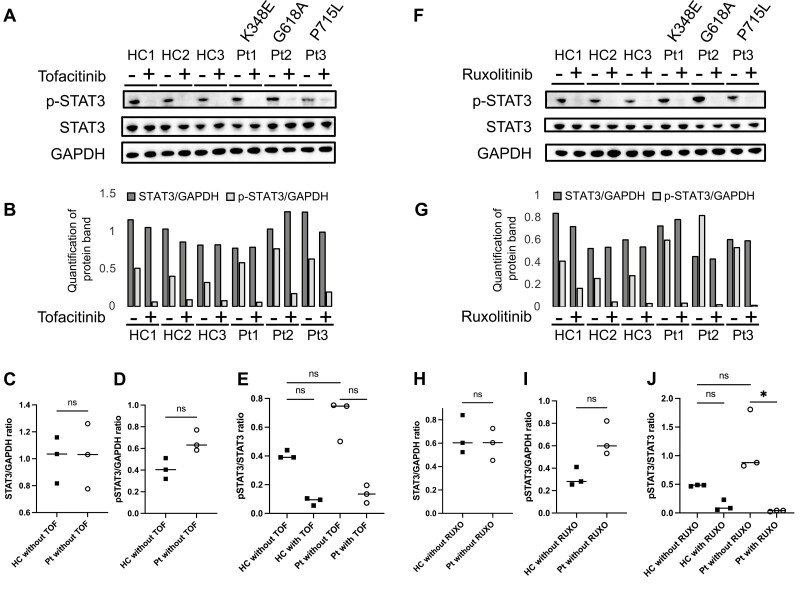
**Western blot analysis of STAT3 phosphorylation.** Western blot analysis of the Epstein-Barr virus transformed lymphoid cell lines (EBV-LCLs) derived from the patients after stimulation with fusion protein of IL-6R and IL-6 (FP6). In addition, the cells were treated with and without either tofacitinib (A) or ruxolitnib (F). (B–E and G–J) Quantification of the protein band intensity (pSTAT3/STAT3 corrected to the GAPDH internal control, and pSTAT3/STAT3 ratio in each sample) was measured using ImageJ software (National Institutes of Health, Bethesda, MD). Asterisks indicate p < 0.05.

Western blot analysis confirmed the increased phosphorylation of STAT3 following FP6 stimulation using EBV-LCLs. The EBV-LCLs from cases 1 (Pt1, p.K348E), 2 (Pt2, p.G618A), and 3 (Pt3, p.P715L) and three healthy controls (HC1–3) were subjected to cell stimulation assays (Fig. 4A, B, F, and G). STAT3 expression levels of the cells from the patients with STAT3-GOF and HCs were comparable (Fig. 4C and H); there were no significant differences in STAT3 phosphorylation between individuals, although an increased trend was seen amongst patients with STAT3-GOF (Fig. 4D, E, I, and J). Co-incubation with 1.0 μM TOF suppressed STAT3 phosphorylation (Fig. 4E), and the suppression of STAT3 phosphorylation was also observed when co-incubated with 1.0 μM RUXO (Fig. 4J). A statistically superior difference in p-STAT3 was found between the RUXO-treated and non-treated groups with respect to the patients’ EBV-LCLs, whereas no superior difference was found between that of the TOF-treated and non-treated groups, although a trend toward lower values was noted in the TOF group (Fig. 4E and J).

## Discussion

We administered JAKi to four Japanese patients with STAT3-GOF and confirmed its clinical efficacy. In addition, cell stimulation assays using EBV-LCLs confirmed that treatment with TOF or RUXO could inhibit STAT3 phosphorylation, which supports the clinical efficacy of the drug.

Inborn errors of immunity (IEIs) are a recently established concept that includes not only primary immunodeficiency disease but also a wide range of diseases, including autoinflammatory and autoimmune diseases. One of the IEIs, STAT3-GOF was first described in 2014 and has since been reported worldwide [7]. As with many other IEIs, allogeneic hematopoietic cell transplantation (HCT) was initially performed as a curative treatment for patients with STAT3-GOF; however, transplantation complications such as infections and graft-versus-host disease often led to patient death or critical adverse events [9, 17]. Thus, a treatment alternative to HCT is desirable.

Tocilizumab, an anti-IL-6 receptor antibody, is a safe treatment option as an HCT replacement. Tocilizumab, which inhibits the IL-6 receptor, a JAK/STAT3 signaling molecule, had previously been administered to several patients and was expected to have efficacy against the symptoms of patients with STAT3-GOF. Although it was partially effective in improving their symptoms such as arthropathy, the therapeutic effect was insufficient [4, 7, 9]. Recently, case reports of patients with STAT3-GOF who received JAKi have been increasing. Furthermore, JAKi has been reported to be effective not only in patients with STAT3-GOF but also in those with STAT1-GOF and other IEIs [7, 9–13, 18–20].

The previous reports of patients with STAT3-GOF that were treated with JAKi are summarized in Figure 1 and Supplementary Table 1. In our four cases, 17 patients were identified. JAKi was often administered to these patients to improve enteropathy or lung diseases, and most were at least partially ameliorated [10, 11, 13–16]. Furthermore, JAKi has been effective for a variety of conditions, including type 1 diabetes [15], delayed development [16], and mucosal dysplasia [13].

In this study, JAKi appeared effective in ameliorating STAT3-GOF symptoms, including polyarthritis, enthesitis, periodic fever, skin rash, liver dysfunction, gastrointestinal symptoms, and lymphadenopathy. Furthermore, regardless of the site of the variants, cell stimulation assays using EBV-LCLs showed that JAKi suppressed STAT3 phosphorylation. This finding supports the idea that JAKi is an effective treatment option for patients with STAT3-GOF.

The results from the cell stimulation assays suggest that RUXO might be a more effective inhibitor of STAT3 phosphorylation than TOF. STAT3 is responsible for signaling through JAK1 and JAK2. RUXO only inhibits JAK1 and JAK2, whereas TOF functions by inhibiting JAK1–3 [2, 21]. Furthermore, TOF has a higher inhibitory activity against JAK1 and JAK2 compared to RUXO; RUXO inhibits JAK1 and JAK2 at IC_50_ values of 6.4 nM and 8.8 nM, respectively, and those of TOF against JAK1 and JAK2 were 15.1 nM and 77.4 nM, respectively [22]. Based on these mechanisms, RUXO might be a more pathognomonic and suitable treatment for patients with STAT3-GOF. However, in clinical practice, RUXO is associated with a higher frequency of adverse events such as myelosuppression and liver dysfunction than that of TOF, therefore determining which is better to use remains controversial.

This study has two limitations. First, this study is limited by the small sample size and retrospective design. Second, our patients were spontaneously treated with other drugs and medical interventions such as prednisolone. Therefore, although the improvements in clinical symptoms and radiological findings are very likely to be due to JAKi, it is not possible to be definitive. In the future, a larger prospective study is expected.

In conclusion, JAKi was effective in patients with STAT3-GOF and various clinical phenotypes. Therefore, JAKi may be an effective treatment option for patients with STAT3-GOF.

## Supplementary Material

ltad027_suppl_supplementary_table

## Data Availability

The datasets generated during and/or analyzed during the current study are not publicly available but are available from the corresponding author on reasonable request.

## Abbreviations

CRP: C-reactive protein
EBV: Epstein-Barr virus
FP6: fusion protein of IL-6R and IL-6
GAPDH: glyceraldehyde-3-phosphate dehydrogenase
GOF: gain-of-function
HC: healthy control
HCT: hematopoietic cell transplantation
IBD: inflammatory bowel disease
IEI: inborn errors of immunity
IL-6: interleukin-6
IP: interstitial pneumonia
JAK: Janus kinase
JAKi: JAK inhibitor
LCL: lymphoid cell line
RUXO: ruxolitinib
STAT3: signal transducer and activator of transcription 3
TOF: tofacitinib
WES: whole-exome sequencing
