# Supplementary material for: Janus kinase inhibitors ameliorate clinical symptoms in patients with STAT3 gain-of-function
Source: Immunother Adv. 2023 Nov 24;3(1):ltad027. doi: 10.1093/immadv/ltad027 (PMC10977912; doi:10.1093/immadv/ltad027)
Supplement: ltad027_suppl_supplementary_table [file ltad027_suppl_supplementary_table.docx]

**Supplement Table 1.** **Clinical features of our patients and a literature review of previously reported STAT3-GOF patients treated with JAK inhibitors**

|  | **Patient No.**  **in Figure 1** | **Patient No. in publication** | **Variants and protein change** | **Age of diagnosis, sex** | **Age of JAKi initiation, agent** | **History of treatment with tocilizmab** | **Hematologic  disorder** | **Endocrine dysfunction** | **Enteropathy** | **FTT** | **Lung disease** | **Lymphoproliferative disease** | **Skin disease** | **CNS**  **disorder** | **Hepatitis** | **Arthritis** | **Others** |
| --- | --- | --- | --- | --- | --- | --- | --- | --- | --- | --- | --- | --- | --- | --- | --- | --- | --- |
| **This study** | 1 | 1 | c.1042A>G, p.K348E | 17 months, M | 3 years, Tofacitinib | No | No | No | Yes | No | No | No | **Atopic dermatitis** | No | No | No | **Periodical fever**, Incomplete KD |
|  | 2 | 2 | c.1853G>C, p.G618A | 8 years,  F | 10 years, Tofacitinib | No | Hypogammaglobulinemia,  Lymphopenia | No | IBD-U | No | No | No | Vitiligo | No | **AIH** | **Multiple enthesitis** | Recurrent AOM |
|  | 3 | 3 | c.2144C>T, p.P715L | 14 years,  F | 14 years, Tofacitinib | Yes | AIN, ITP, Hypogammaglobulinemia | No | No | No | No | **Lymphadenitis** | No | No | No | **Polyarthritis, joint deformity** | Recurrent AOM |
|  | 4 | 4 | c.1244A>G, p.E415G | 4 years,  F | 7 years, Ruxolitinib | Yes | **Hypogammaglobulinemia** | Hypothyroidism, IDDM | **Enteritis with anti-villin antibody positive** | **Yes** | Recurrent respiratory infection, **ILD** | No | No | No | **NAFLD** | No | No |
| **Forbes, et al. [11]** | 5 | 12 | c.454C>T, p.R152W | N.D.,  M | 8 years, Ruxolitinib | Yes | Anemia | No | **Yes** | Yes | ILD | **Hepatosplenomegaly** | N.D. | N.D. | **Yes** | No | **Portal hypertension** |
|  | 6 | 13 | c.521T>C, p.F174S | N.D.,  F | 14 years, Ruxolitinib | Yes | Anemia, neutropenia, thrombocytopenia, DIC | Hypothyroidism | GI bleeds | Yes | Pulmonary hemorrhage | Hepatosplenomegaly | No | N.D. | AIH | No | No |
|  | 7 | 14 | c.857A>G, p.E286G | N.D.,  M | 3 years, Ruxolitinib | Yes | No | No | **Yes** | Yes | No | Hepatosplenomegaly, **HLH** | No | N.D. | No | No | No |
|  | 8 | 15 | c.1032G>C, p.Q344H | 4 years,  F | 8 years, Ruxolitinib | Yes | **AIHA** | No | **Yes** | **Yes** | **ILD** | Hepatosplenomegaly | No | N.D. | AIH | No | No |
|  | 9 | 16 | c.1261G>A, p.G421R | N.D.,  M | 13 years, Tofacitinib | Yes | AIHA, thrombocytopenia | No | No | Yes | No | Hepatosplenomegaly | **Scleroderma** | N.D. | AIH | **Polyarthritis** | No |
|  | 10 | 17 | c.2144C>T, p.P715L | N.D.,  M | 15 years, Ruxolitinib | Yes | Thrombocytopenia | Hashimoto’s thyroiditis | Yes | **Yes** | **ILD** | Lymphadenitis, splenomegaly | Eczematous dermatitis | N.D. | No but had NRH | No | Portal hypertension |
| **Parlato, et al. [18]** | 11 | 1 | c.1201A>G, p.N401D | 25 years,  F | 25 years, Ruxolitinib | Yes | Neutropenia, ITP, Hypogammaglobulinemia | Hashimoto’s thyroiditis | **Yes** | Yes | N.D. | N.D. | N.D. | N.D. | N.D. | N.D. | Recurrent  CD infections |
| **Silva-Carmona, et al. [19]** | 12 | 1 | N.D.,  p.E415L | 7 years,  F | 7 years, Ruxolitinib | Yes | Hyper-IgA,  B-cell deficiency | N.D. | Yes | Yes | **ILD** | Hepatosplenomegaly | Atopic dermatitis | N.D. | N.D. | Oligoarthritis | Dental abnormalities |
|  | 13 | 2 | c.1178T>C, p.V393A | 21 years,  F | 21 years, Tofacitinib | Yes | No | Delayed puberty | Enteritis with anti-DGP antibody positive | Yes | **ILD** | No | No | Deafness | No | Polyarthritis,  joint deformity | No |
|  | 14 | 4 | N.D.,  p.M329K | 17 years,  N.D. | 18 years, Tofacitinib | No | N.D. | N.D. | N.D. | N.D. | **ILD** | N.D. | N.D. | N.D. | N.D. | N.D. | No |
| **Wegehaupt, et al. [20]** | 15 | 1 | c.2144C>T, p.P715L | 17 months,  M | 17 months, Ruxolitinib | No | No | **IDDM (insulin demand)** | No | No | No | **Lymphadenitis** | **Panniculitis** | No | No | No | Lipodystrophy |
| **Sarfati, et al. [21]** | 16 | 1 | c.1973A>T, p.K658M | 12 months,  M | 12 months, Ruxolitinib | Yes | N.D. | IDDM | **Yes** | **Yes** | **ILD** | N.D. | N.D. | **DD**, epilepsy | No | No | No |
| **Mulvihill, et al.  [22]** | 17 | 1 | c.1260T>G, p.N420K | N.D.,  F | 14 years, Ruxolitinib | No | No | No | No | No | No | No | Eczematous dermatitis | No | No | No | **Oral mucosal dysplasia** |

Symptoms highlighted in underbar indicate presented symptoms when JAKi was initiated.

AIH, autoimmune hepatitis; AIHA, autoimmune hemolytic anemia; AIN, autoimmune neutropenia; AOM, acute otitis media; CD, *Clostridium Difficile*; CNS, central nervous system; DD, Developmental delay; DGP, Deamidated Gliadin Peptide; GI, gastrointestinal; FTT, Failure to thrive; HLH, hemophagocytic lymphohistiocytosis; ILD, interstitial lung disease; IBD-U, inflammatory bowel disease unclassified; ITP, immune thrombocytopenic purpura; JAK, Janus kinase; JAKi, JAK inhibitors; KD, Kawasaki disease; NAFLD, nonalcoholic fatty liver disease; N.D., No data; NRH, nodular regenerative hyperplasia; T1DM, type 1 diabetes mellitus
